# Adverse Childhood Experiences and Adolescent Delinquency: A Theoretically Informed Investigation of Mediators during Middle Childhood

**DOI:** 10.3390/ijerph20043202

**Published:** 2023-02-11

**Authors:** Dylan B. Jackson, Melissa S. Jones, Daniel C. Semenza, Alexander Testa

**Affiliations:** 1 Department of Population, Family, and Reproductive Health, Johns Hopkins Bloomberg School of Public Health, Baltimore, MD 21205, USA; 2 Sociology Department, College of Family, Home, and Social Sciences, Brigham Young University, Provo, UT 84602, USA; 3 Department of Sociology, Anthropology, and Criminal Justice, Rutgers, Camden, The State University of New Jersey, 405-7 Cooper Street, Camden, NJ 08102, USA; 4 Department of Management, Policy and Community Health, University of Texas Health Science Center at Houston, Houston, TX 77030, USA

**Keywords:** adverse childhood experiences, delinquency, childhood, adolescence, United Kingdom

## Abstract

Objective: The purposes of this study are twofold. First, we explore the associations between cumulative ACEs at ages 5 and 7 and delinquency at age 14 in a national sample of youth in the United Kingdom (UK). Second, we explore the role of five theoretically relevant mediators in explaining this relationship. Methods: Analyses were based on data from the UK Millennium Cohort Study—a prospective, longitudinal birth-cohort study of more than 18,000 individuals in the United Kingdom. Results: The results indicate that early ACEs are significantly associated with adolescent delinquency, with effects becoming significantly larger as ACEs accumulate. Findings also reveal that child property delinquency, substance use, low self-control, unstructured socializing, and parent–child attachment at age 11 all significantly mediate the relationship between early ACEs and delinquency in adolescence, with early delinquency and low self-control emerging as the most robust mediators. Conclusions: Findings point to a need for early ACEs screening and a Trauma-Informed Health Care (TIC) approach in early delinquency prevention efforts. Early intervention efforts that bolster child self-control and curtail early-onset problem behaviors may also disrupt pathways from ACEs to adolescent delinquency.

## 1. Introduction

Adverse childhood experiences (ACEs), including abuse, neglect, and living in a dysfunctional household environment, can produce an array of negative life outcomes in both the short- and long-term [1,2,3,4]. In the U.S., ACEs are also quite common, with nearly two-thirds of adults reporting exposure to ACEs and about 25% reporting exposure to three or more [5,6,7]. Such cumulative exposure is particularly harmful, given the evidence of a dose–response relationship in which greater exposure to more and varied ACEs leads to even poorer outcomes [8,9]. Although the prevalence of certain ACEs like abuse and neglect has declined in the 21st century, other harmful exposures (e.g., parental drug and alcohol use) have increased, necessitating a deeper understanding of the deleterious effects of ACEs [10].

In recent years, research has identified adolescent delinquency as an important negative repercussion of greater ACE exposure [11,12,13,14,15], highlighting the importance of ACEs prevention programs, proactive screening, and early-childhood interventions in developmental crime prevention efforts [2,5]. Even so, there remains at least four key limitations to this research. First, research on ACE exposure and adolescent delinquency has yet to assess how mediating factors during middle childhood (approximately ages 6–12) inform this relationship using a time-ordered, longitudinal approach. Although researchers have examined mediating pathways through mechanisms such as strain-induced negative emotionality [15], many criminologically relevant pathways related to child misbehaviors, parental relationships, and types of childhood socialization are rarely explored. Furthermore, to our knowledge, researchers have yet to disentangle the relevance of multiple *concurrent* theoretical mediators to explain the ACEs–delinquency link. As such, there remains limited theorizing around how ACE exposure increases the risk for delinquency through multiple, theoretically relevant mediating pathways. Second, and relatedly, most research relies on retrospective accounts of ACE exposure and delinquency among older adolescents or adults, making it challenging for researchers to test relevant mediating mechanisms. Third, the majority of studies conducted on the relationship between ACE exposure and adolescent delinquency rely on samples where most or all of the youth have had formal contact with the criminal justice system [11,15,16,17]. Relatively few criminological studies have examined how ACEs impact delinquency among non-justice-involved populations [13,14]. Finally, the vast majority of the research in this area has been conducted in the United States, making it difficult to generalize findings on ACEs and delinquency to other national contexts.

To address these gaps in the literature, we leveraged a longitudinal birth-cohort study of more than 18,000 individuals in the United Kingdom. Using caregiver reports of ACEs from early childhood (among children ages five and seven), we analyzed how different levels of ACE exposure were associated with adolescent delinquency. We then investigated the role of multiple mediating mechanisms during middle childhood derived from criminological theory and research, including early delinquency and substance use, low self-control, peer socializing patterns, and parent–child bonding. This study contributes to a broader understanding of how ACEs influence the future risk of adolescent delinquency in a non-institutionalized population outside of the United States through criminologically relevant pathways during middle childhood. Our results have implications for reducing delinquency that emphasize early and middle childhood intervention as well as service provision for children exposed to adverse conditions. 

## 2. Adverse Childhood Experiences and Delinquency

Research has shown that ACEs are harmful for an array of short- and long-term outcomes [3,9], including engagement in crime and delinquency [11,12,13,14,15]. Although ACE exposure occurs during all stages of childhood, it is most common during early (5 years or younger) and middle childhood (6 to 12 years) compared to adolescence [18]. Further, there is a documented graded effect which shows that the more ACEs a child experiences, the greater the risk of adverse outcomes later in life [8]. Many children experience ACEs across numerous contexts [19,20], suggesting that a small subset of disproportionately exposed children is particularly vulnerable to negative outcomes as they develop.

A dose–response dynamic between ACE exposure and delinquency has been increasingly documented across numerous studies [11,16,21,22,23]. Youth who have experienced more ACEs are more likely to engage in violent offending [16], gang involvement [24], substance use [21,24], and general delinquency [17]. In a recent systematic review, Graf and colleagues [2] found a consistent and graded positive relationship between ACEs and juvenile justice contact, regardless of the type of justice system contact and geographic region of the U.S. Furthermore, youth who are incarcerated and have been exposed to more ACEs are at greater risk for reoffending and recidivism [15,25]. Most of this research has been conducted in the United States, although the link between ACE exposure and delinquency is increasingly being documented in other countries such as Italy [26], Portugal [27], and Germany [28]. 

One criminological theory that researchers have leveraged to explain the connection between ACEs and delinquency is Agnew’s [29] general strain theory [12,15]. General strain theory (GST) posits that individuals undergo strains such as the inability to achieve one’s goals, the experience of something harmful, or the removal of something valued, which then lead to negative emotions (e.g., anger or frustration) and, in certain instances, delinquent coping. While the negative emotions that result from ACE exposure may lead to short-term delinquent coping, they may also compound and increase the risk of delinquency over time [30,31]. The strain and stress of ACE exposure can even “get under the skin” by heightening allostatic load and generating chronic fight or flight sensations associated with a heightened risk of delinquent and violent behavior [32]. Ultimately, exposure to ACEs results in persistent dysregulation of neuroendocrine responses to stress and strain that can have meaningful implications for later delinquency.

The predominant focus of criminologists on employing a general strain framework to explain the ACEs–delinquency connection, while understandable, has obscured the possible relevance of alternative mediating mechanisms during childhood. Namely, the harms of ACEs may not only engender strain-induced negative emotionality among children but may also impact their social, cognitive, and behavioral development as they grow into adolescents. Although ACE exposure may influence the risk of delinquency through the long-term damages of trauma, increased stress, and the heightened negative emotions it can engender, we argue there are important theoretical considerations yet to be explored that deserve more attention, particularly during middle childhood. We therefore focus on five mediating mechanisms in this study: child delinquency, child substance use, child low self-control, unstructured socializing with peers, and parental bonding. Explicitly, we consider how ACE exposure in early childhood corresponds to heightened delinquency in adolescence via each of the mediating mechanism in middle childhood. 

## 3. The Potential Role of Multiple Mediators in Middle Childhood

### 3.1. Childhood Delinquency and Substance Use

Most research on ACE exposure and delinquency focuses on early childhood exposure and its implications for delinquent behavior in adolescence without sufficient attention to the intervening years of middle childhood. Childhood behavioral problems represent some of the most substantial predictors of delinquency through adolescence and into adulthood [33,34,35,36]. It follows, then, that if ACE exposure increases the risk of problem behaviors in middle childhood, then the child will be more likely to engage in delinquency during adolescence. As alluded to in criminological theoretical frameworks [37], if younger children engage in behaviors like substance use or minor property offenses, these behaviors may persist and even escalate into adolescence, particularly if they remain exposed to the same harmful home environments and/or do not have the benefit of professional intervention.

There is relatively little delinquency-focused prospective research that examines the short-term behavioral impacts of ACEs in middle childhood for children exposed when they are very young. Most of the literature on ACEs and delinquency is either retrospective or focuses on longitudinal effects without attention to proximal outcomes in middle childhood. From a life-course perspective, adolescents exposed to ACEs in early childhood may be more likely to engage in adolescent delinquency not only due to experiences of latent trauma, compounded stress, and emotional harm but because those experiences catalyze early-onset misconduct during childhood. 

The available research on the subject generally shows that greater exposure to ACEs in early childhood increases the risk for externalizing problems in middle childhood [38,39,40]. For example, ACEs in infancy and toddlerhood were found to correspond to externalizing problems in middle childhood in a demographically diverse sample of low-income families as part of the Early Head Start impact study [40]. Hunt and colleagues [39] similarly found that ACEs among children under five were strongly associated with externalizing behaviors in middle childhood using data from the Fragile Families and Child Well-Being Study (FFCWS). Additionally, recent research focusing on youth with histories of justice system involvement has underscored the relevance of substance use as a mediator of the ACEs–delinquency link, despite its lack of focus on middle childhood [24]. Still, the literature collectively provides good reason to anticipate that middle childhood misconduct and substance use may at least partially explain associations between early ACE exposure and adolescent delinquency. 

### 3.2. Low Self-Control

Low self-control is one of the most robust predictors of adolescent delinquency identified by criminologists [41,42,43,44], and research has demonstrated that ACE exposure and low self-control share similar negative behavioral and health-related consequences over time [45]. It is therefore plausible that early exposure to adverse conditions such as abuse, neglect, or household dysfunction influences self-control in middle childhood with later implications for delinquency during adolescence. For instance, Meldrum and colleagues [45] found that ACE exposure corresponds with reductions in low self-control using two independent samples from Michigan and Florida. The findings revealed that maltreatment-related ACEs are particularly influential for self-control compared to household-dysfunction-related ACEs. Recent research using data from the Fragile Families and Childhood Wellbeing study has also revealed that as ACEs increase, self-control declines for both boys and girls [46,47].

According to self-control theory [41], the primary predictor of low self-control is ineffective parenting. Children who experience high ACE exposure are likely to be victimized at the hands of parents, receive little behavioral monitoring, and receive an improper balance of rewards and punishments, all of which reduce self-control early in life [46]. Living in a dysfunctional household may also expose children to highly stressful environments where family members exhibit low self-control by yelling, acting violently, or fighting with one another. This may lead to children modeling or imitating these negative coping behaviors, leading to greater internalization of strategies that solidify their own deficits in self-control [45]. Parents or guardians may also simply be less present in the home, leading children to have to fend for themselves more frequently without the proper guidance to learn the necessary strategies to improve self-control. For all these reasons, children exposed to ACEs in early childhood, a critical period for the development of self-control, may experience reductions in self-control that influence delinquent behaviors into adolescence. 

### 3.3. Unstructured Socializing with Peers

Beyond self-control and childhood delinquency, there are critical aspects of socialization that may also influence the relationship between early ACE exposure and adolescent delinquency. First, peer relationships and socialization patterns represent important predictors of delinquency that may be shaped by early traumatic experiences in the home. Spending time with friends without a common structured activity in the absence of an adult authority figure (i.e., unstructured socializing) is a strong predictor of both adolescent delinquency and substance use [48,49,50,51]. Children who grow up in a chaotic environment or experience victimization at the hands of a family member early in life may be more likely to be exposed to negative peer influences in middle childhood. As they gain and develop more independence, these children may also elect to spend more time away from the watchful eye of parents, or parents may become less vigilant in monitoring their children to the extent that co-occurring stressors/adversities in their own lives overwhelm their capacity to do so. In this manner, ACE exposure may at least partially underpin the formation of early, peer-involved opportunities for misbehavior that may set the stage for later adolescent delinquency.

As Stritzel [52] notes, youth exposed to ACEs may seek out friends with similar negative life experiences, experience greater neighborhood mobility which increases exposure to delinquent peer networks, and may be less likely to be monitored by parents when they are socializing with friends. Each of these heightens the risk that children exposed to ACEs will engage in unstructured socializing with their friends, which may have an enduring influence as the child continues towards adolescence. Empirical research on the longitudinal relationship between peer socializing and delinquency suggests that more time spent involved in unstructured or unsupervised activities in middle childhood sets the stage for greater delinquent behavior in adolescence. For instance, Lam and colleagues [50] found that unsupervised time, especially with mixed or opposite sex peers, in middle childhood longitudinally predicted problem behaviors in adolescence. Conversely, more time spent in supervised activities with those same peers predicted better school performance. In another study of 11 to 17-year-olds, Janssen and colleagues [53] found that the less parental monitoring and supervision enhances the ability of unstructured socializing in criminogenic, highly disordered settings to increase adolescent delinquency. Taken together, the large body of research on unstructured socializing and delinquency in general, as well as the specific research on this topic in middle childhood, suggests that unstructured socializing with peers may represent an important additional mechanism connecting ACE exposure and adolescent delinquency.

### 3.4. Parental Bonding

Finally, children exposed to ACEs early in life are a much greater risk for suffering from strained relationships and decreased attachment to their parents [54,55,56]. Parents may be directly responsible for the abuse or neglect of the children, irreparably damaging feelings of connection and bonding that persist as the child ages into middle adulthood and adolescence. Children exposed to ACEs may also lack a strong relationship with a parent if the parent has been preoccupied or absent as a result of substance use, mental illness, or time away while incarcerated [45]. Children of divorced parents may similarly struggle to feel connected to one or both of their parents if they are living away from one of them or forced to split their time [57]. ACEs have significant potential to tax child–parent bonds, which may have significant implications for problem behaviors and delinquency as the child moves into adolescence. 

Social bonds with parents, particularly attachment, are especially important for the risk of delinquent behavior of offspring [58,59,60,61]. Youth who have a greater connection with their parents are less likely to engage in delinquency, whereas youth who suffer from strained parental bonds are at higher risk for delinquent behaviors [62]. This effect for parents is uniquely non-linear such that individuals with negative attachments to their parents are much more delinquent than those that are neutrally or positively attached [63]. The general attachment–delinquency dynamic holds true for both boys and girls, although stronger effects have been found for attachment to mothers than attachment to fathers [61]. The link between attachment and delinquency is especially strong when children are young and weakens during adolescence. As such, ACE exposure in early childhood may be particularly damaging to parental attachment during more proximate stages of development such as middle childhood, which can then influence the risk of delinquency during emergent adolescence. 

## 4. The Current Study

In summary, prior research on the relationship between ACE exposure and adolescent delinquency, while largely focusing on the strain associated with ACEs, has yet to account for important mediating mechanisms in middle childhood derived from a broader swath of criminological theory and research. We highlight five of these potential pathways, including child property delinquency, child substance use, child low self-control, child unstructured socializing, and parental attachment. As outlined above, greater ACE exposure in early childhood is likely to influence each of these important factors in middle childhood, which then may operate to shape the risk of delinquency in adolescence. 

In the present study, we aimed to address the shortcomings of prior research by drawing on a national, prospective, birth-cohort study of youth from the United Kingdom (UK) Millennium Cohort Study (MCS). In the UK, roughly 8.8% of girls and 9.3% of boys have been found to experience three or more ACEs by age 14 [64]. This is slightly lower than estimates of similar-aged adolescents in the U.S. (~13.1% of 12–17-year-olds have experienced three or more ACEs) [65]. Even so, national comparisons on the ACEs–adolescent delinquency nexus are lacking. Thus, beyond the current study’s focus on the first years of life, early development, and identifying meaningful pathways from ACEs to adolescent delinquency, it is among the first to assess the ACEs–adolescent delinquency nexus among a national sample of youth in the UK, which informs the cross-population generalizability of this association. Previous UK studies using local or regional samples have indeed intimated positive associations between ACEs and various acts of violence and misconduct among young people [66,67]. The current study builds upon this work by using a recent, nationally representative longitudinal cohort study to test these associations. Specifically, we posit the following two research questions:

1. Is the accumulation of adverse childhood experiences (ACEs) at ages 5 and 7 associated with delinquency at age 14 among youth in the UK?
2. Do child property delinquency, substance use, low self-control, unstructured socializing, and connectedness to parents at age 11 mediate any association between ACEs and adolescent delinquency among these youth?

## 5. Data & Methods

Data from the UK MCS were employed in the current study. The MCS is a national, longitudinal study of 18,818 children born in the UK between 2000 and 2002. To date, data have been collected at seven intervals (labelled Sweeps, henceforth noted as “S”) when children were approximately 9 months of age (S1, 2001), 3 years of age (S2, 2004), 5 years of age (S3, 2006), 7 years of age (S4, 2008), 11 years of age (S5, 2012), 14 years of age (S6, 2015), and 17 years of age (S7, 2018). The present study employed data from S2 through S6, with covariates being derived from S2, ACEs being derived from S3 and S4, mediators being derived from S5, and delinquency being derived from S6. At S6 (when youth were 14 years of age), 11,313 youth participated in the young person questionnaire. The present study was restricted to the sample of youth who participated in the S6 young person questionnaire and had valid data on all delinquency items (*N* = 11,192). The MCS data were obtained using a stratified cluster sampling design, with the population being stratified by UK country—England, Wales, Scotland, and Northern Ireland. The study oversampled children from families living in disadvantaged areas (i.e., the poorest 25% of wards from the ward-based Child Poverty Index) and in those with higher proportions of ethnic minority groups (wards that had an ethnic minority indictor of at least 30%). Thus, the final sample included a higher number of children and families at risk of various forms of adversity and hardship (relative to the general population), which makes the data well-suited to our research question. Even so, the data contain the appropriate sampling weights to produce findings that are nationally representative and generalizable to the UK as a whole. For additional details on the study design and sample, see https://cls.ucl.ac.uk (access date 26 November 2022). 

### 5.1. Adolescent Delinquency

At S6, the young person questionnaire was administered to youth participants, which asked a series of questions pertaining to adolescent delinquency [68,69]. This series of questions included 11 items pertaining to delinquent activities in which the youth may have engaged in the 12 months prior to the survey. Specifically, youth were asked the following questions: *In the last 12 months* (1) Did you ever stay away overnight without your parents knowing where you were? (2) Have you been noisy or rude in a public place so that people complained or got you in trouble? (3) Have you taken something from a shop without paying for it? (4) Have you written things or spray painted on a building, fence, train, or anywhere else where you shouldn’t have? (5) Have you on purpose damaged anything in a public place that didn’t belong to you, for example by burning, smashing or breaking things like cars, bus shelters and rubbish bins? (6) Have you pushed or shoved/hit/slapped/punched someone? (7) Have you used or hit someone with a weapon? (8) Have you stolen something from someone. e.g., a mobile phone, money etc.? (9) Have you accessed, or hacked into, someone else’s computer, e-mail or social networking account without their permission? (10) Have you used the internet to send viruses, or other harmful software, to deliberately damage or infect other computers? and (11) How many times have you had five or more alcohol drinks at a time? Items 1 through 10 included the response options *Yes* (1) and *No* (0). Item 11 included the response options *Never* (0), *1–2 times* (1), *3–5 times* (2), *6–9 times* (3), and *10 or more times* (4). However, item 11 was dichotomized (i.e., never = 0, else = 1) for the purposes of this study (and given its zero-inflation and positive skew). Items were summed into a count measure (ranging from 0–11) capturing the variety of delinquent activities in which youth engaged at S6 of data collection (Kuder–Richardson alpha = 0.70). 

### 5.2. Adverse Childhood Experiences (ACEs)

To measure ACEs using the MCS data, we followed the lead of Straatmann and colleagues [70] and included seven indicators of ACEs: *Verbal Maltreatment*, *Physical Maltreatment*, *Parental Divorce*, *Parental Mental Illness*, *High Frequency of Parental Alcohol Use*, *Domestic Violence*, and *Parental Drug Use*. As noted by Straatmann in previous MCS research [70], these ACEs are commonly used in other studies, which can facilitate useful comparisons [3,71,72]. For the purposes of this study, these items were extracted from S3 and S4 of the MCS study, when children were on average five and seven years old, respectively (with the exception of *Parental Drug Use*, which was only available at S3). Children were considered to have experienced an ACE if the main caregiver respondent (usually the mother, ~98%) indicated the presence of a given ACE at *either* S3 or S4. All items were taken from the S3 or S4 Computer-Assisted Person Interview (CAPI) Questionnaire, as completed by the main caregiver. For measurement details pertaining to each of the seven ACEs, see the Appendix A.

### 5.3. Mediators (S5)

We examined the following five mediators derived from S5, when children were on average 11 years of age: child property delinquency, child substance use, child low self-control, child unstructured socializing, and parent–child attachment.

***Child Property Delinquency***. Given that prior delinquency is a notable risk factor for future delinquency, it was essential to account for early delinquent behavior in our mediating models. Doing so is also critical when examining multiple correlated mediators to ensure that pathways from ACEs to adolescent delinquency are not fully explained by earlier engagement in delinquency at a time when it is less developmentally expected. In line with prior research [73,74], we constructed a binary item derived from the S5 child self-completion questionnaire regarding whether youth had participated in any of the following three activities in their final year of primary school (i.e., ~11 years of age): (1) taken something from a shop without paying for it; (2) written things or sprayed paint on a building, fence, train, or anywhere else where you shouldn’t have; and/or (3) on purpose damaged anything in a public place that didn’t belong to you, for example by burning, smashing or breaking things like cars, bus shelters and rubbish bins? Due to the low rates of property delinquency at this age, and in accordance with prior research [73,74], we collapsed all three items into a single binary indicator of child property delinquency (S5). 

***Child Substance Use***. Also in line with prior research [73,74], we constructed a binary item derived from the S5 child self-completion questionnaire regarding whether youth had participated in either of the following two activities in their final year of primary school (i.e., ~11 years of age): (1) tried a cigarette (even if it was only a single puff); and/or (2) had an alcohol drink (more than a few sips). Due to the low rates of substance use at this age, and in accordance with prior research [73,74], we collapsed these two items into a single binary indicator of child substance use (S5). 

***Child Low Self-Control***. In order to capture children’s level of self-control during late childhood (S5), we employed five items utilized in recent MCS research [75]. The items were derived from the hyperactivity/inattentive subscale of the Strength and Difficulties Questionnaire (SDQ), a scale with good reliability that has been widely validated as a screening tool to capture self-regulatory difficulties. At S5, main caregivers were asked in the main household questionnaire about how well the following statements described their child, after being prompted with “Please give your answers on the basis of [CHILD]’s behavior over the last six months”: (1) [Child] is restless, overactive, cannot stay still for long; (2) [Child] is constantly fidgeting or squirming; (3) Child is easily distracted, concentration wanders; (4) [Child] thinks things out before acting (reverse-coded); and (5) [Child] sees tasks through to the end, good attention span (reverse-coded). Response options included *Not true* (1), *Somewhat true* (2), and *Certainly True* (3), with items 4 and 5 being reverse-coded. Items were averaged into an index, with higher scores reflecting low levels of self-control during late childhood (alpha = 0.80). Notably, these items from the SDQ were employed as indicators of self-control in recent criminological research [76].

***Child Unstructured Socializing***. To capture unstructured socializing, we employed child responses to the following three questions from the S5 child self-completion questionnaire: (1) When you are not at school, how often do you spend time with your friends?; (2) At the weekend, how often do you spend time with your friends, but without adults or older children, doing things like playing in the park, going to the shops, or just “hanging out”?; and (3) In the afternoon after school, how often do you spend time with your friends, but without adults or older children, doing things like playing in the park, going to the shops, or just “hanging out”? Response options for items 1 and 3 include *never* (0), *less often than once a month* (1), *at least once a month* (2), *at least once a week* (3), and *most days* (4). Response options of item 2 include never (0), less often than once a month (1), at least once a month (2), and most weekends (3). Due to disparate response options, scores were standardized before averaging them into an index (alpha = 0.83). 

***Parent–Child Attachment***. Finally, in order to capture parent–child attachment, we employed an item from the main household questionnaire at S5. Main caregivers were asked, “Overall, how close would you say you are to [CHILD]?”. Response options included *not very close* (1), *fairly close* (2), *very close* (3), and *extremely close* (4). Thus, higher scores are indicative of a closer parent–child relationship at S5. 

### 5.4. Covariates

The following covariates were included in the multivariate models to address omitted variable bias and minimize the likelihood of spurious results: *Youth Age* (in years; S6), *Sex* (Male = 1; S6), *Race* (Asian, Black, Mixed, and Other, with White as reference category; S6), *Mother’s Age at Birth* (S2), *Parent Education* based on National Vocational Qualification (NVW; NVQ 1 [reference], NVQ 2, NVQ 3, NVQ 4, NVQ 5, overseas or other education, and none; S2;) [73], *Household Poverty* (McClements below 60% median poverty indicator; S2) [77], *Household Size* (i.e., number of persons residing in focal child’s household; S2), and *Low Neighborhood Safety* (i.e., parent perceptions of safety of their neighborhood from very safe [1] to very unsafe [5]; S2). 

### 5.5. Plan of Analysis

The analysis proceeded as follows. First, we calculated descriptive statistics pertaining to the full analytical sample of youth (*N* = 11,192). Second, we estimated unadjusted and adjusted zero-inflated negative binomial regression models of the association between ACEs and adolescent delinquency. Zero-inflated negative binomial regression was employed given that the outcome variable (i.e., adolescent delinquency) is not only a count measure, but is also both zero-inflated and overdispersed. Thus, use of alternative modeling strategies (e.g., OLS or Poisson) would result in biased estimates [78]. Third, we employed the Karlson–Holm–Breen (KHB) method to examine the extent to which five variables of interest from S5 (i.e., child property delinquency, child substance use, child low self-control, child unstructured socializing, and parent–child connectedness) mediated associations between ACEs and adolescent delinquency [79]. We opted to test mediation using the KHB method for two reasons. First, because we simultaneously considered multiple, correlated mediating variables, the KHB method provides the benefit of (1) decomposing the independent mediating effects of each of these individual variables and (2) calculating whether the change in the focal independent variable across models—following the inclusion of that mediator—is greater than excepted by chance. Second, coefficients across nested nonlinear models cannot be directly compared because of a rescaling of the model that occurs after additional variables are added. The KHB method is able to adjust for this rescaling and provides an estimate of the proportion of the association between the independent and dependent variables that is explained by a given mediator. For these reasons, this approach to testing mediation has grown in popularity—including among criminologists—over the past several years [68,80,81]. All analyses were conducted in STATA v 17.1 (StataCorp, College Station, Texas) using multiply imputed data (chained equations, 20 imputations), and weights were employed to adjust for nonresponse, probability of selection, and the demographic distribution of the target population.

## 6. Results 

We begin by examining the results of the univariate descriptive analyses, which are displayed in Table 1. The findings indicate that the average score on the adolescent delinquency count outcome was 0.75, with a standard deviation of 1.20. Just under 57.83% of youth reported zero delinquent acts, whereas 25.33% of youth reported only one type of delinquent act. The remaining youth (~17%) reported engagement in two or more forms of delinquency. In terms of parent-reported ACEs at ages five (S3) or seven (S4), 38.09% reported no exposure, 44.66% reported a single exposure, 14.34% reported exposure to two ACEs, and 2.91% reported exposure to three or more ACEs. Youth were on average 13.77 years of age at S6 and 49.25% were male. The majority of youth were white (79.90%), followed by Asian (10.91%) and mixed race (4.71%). Mothers were on average 29.10 years old at the time of the child’s birth, and 31.60% of families fell into the current household poverty categorization. The average household size was 4.22 individuals, and the average low neighborhood safety score was 1.83. In terms of mediators, 7.45% of youth reported property delinquency at S5, and 12.54% reported substance use at S5.

Next, we estimated unadjusted and adjusted zero-inflated negative binomial regression models of the association between ACEs and adolescent delinquency. The findings, which are displayed in Table 2, indicate that accumulating ACEs were significantly associated with higher rates of adolescent delinquency. To be precise, the rate of adolescent delinquency among children reporting one ACE was 21% higher than the rate of delinquency among children reporting no ACEs (*p* < 0.01). Associations were somewhat stronger as ACEs accumulated. For instance, the rate of adolescent delinquency among children reporting two ACEs was 45% higher than the rate of delinquency among children reporting no ACEs (*p* < 0.01), and the rate of adolescent delinquency among children reporting three or more ACE was 66% higher than the rate of delinquency among children reporting no ACEs (*p* < 0.01). Ancillary analyses examining specific ACEs revealed that *Verbal Maltreatment* (IRR = 1.29; CI = 1.20–1.38), *Physical Maltreatment* (IRR = 1.46; CI = 1.18–1.80), *Parental Divorce* (IRR = 1.11; CI = 1.01–1.23), *Domestic Violence* (IRR = 1.36; CI = 1.15–1.60), and *Parental Drug Use* (IRR = 1.97; CI = 1.50–2.59) were also individually associated with adolescent delinquency. In follow-up adjusted analyses of cumulative ACEs, associations were slightly attenuated but remain statistically significant across the board. Additionally, adjusted models revealed that older youth (IRR = 1.22; CI = 1.12–1.32; *p* < 0.01), male youth (IRR = 1.55; CI = 1.14–1.66; *p* < 0.01), mixed-race youth (IRR = 1.17; CI = 1.01–1.35; *p* < 0.05), and youth living in poverty (IRR = 1.14; CI = 1.05–1.35; *p* < 0.01) exhibited significantly elevated rates of delinquency, whereas Asian youth (IRR = 0.72; CI = 0.62–0.85; *p* < 0.01) and youths whose mothers were older at their birth (IRR = 0.98; CI = 0.97–0.99; *p* < 0.01) exhibited significantly lower rates of delinquency.

Finally, we estimated mediation effects by employing the Karlson–Holm–Breen (KHB) method. Specifically, we investigated the extent to which five variables of interest from S5 (i.e., child property delinquency, child substance use, child low self-control, child unstructured socializing, and parent–child attachment) mediated associations between ACEs and adolescent delinquency. The findings are displayed in Table 3. Overall, the results indicate that collectively, the mediators explained anywhere from 40.37–50.49% of the associations between ACEs and adolescent delinquency. All mediators emerged as statistically significant in every instance with one exception: child unstructured socializing did not significantly mediate associations between three or more ACEs and adolescent delinquency. In general, the pattern of results revealed child property delinquency (10.38–20.39% mediation) and child low self-control (10.88–16.49%) to be the most robust mediators, followed by child substance use (6.34–11.26%). Comparatively, parent–child attachment (3.04–8.25%) exhibited somewhat weaker mediation effects, despite being consistently statistically significant. Additionally, unstructured socializing also exhibited relatively weak mediation effects (2.31–5.95%), which fell from significance in the case of three or more ACEs.

## 7. Conclusions

This study examined the relationship between exposure to early ACEs and delinquency among adolescents in the UK. Our first aim was to examine whether accumulating ACEs at ages 5 and 7 were associated with delinquency at age 14 among adolescents. The results echoed prior research, indicating that early ACEs are significantly associated with adolescent delinquency, supporting a GST theoretical framework [29]. Moreover, GST posits that strains that are high in magnitude, long in duration, recent, and frequent can increase the likelihood that strains or stressors will result in criminal coping behaviors. According to Agnew [29], exposure to multiple or repeated strains, like ACEs, may lead to a threshold effect whereby the accumulation of stressors over time produces even more negative outcomes. Our current study’s findings support this supposition. Specifically, we found that as the number of early ACEs incrementally increased, the likelihood of reporting delinquent behaviors also increased. This finding supports prior research on the dose–response relationship between ACEs and delinquency and demonstrates that young children who are exposed to a greater number of ACEs tend to have a higher risk of involvement in delinquency compared to children who report no ACEs [11,13,14,15,16]. 

Additionally, our findings underscore the importance of considering the timing of ACE exposure (here looking at very early ACEs) in later life outcomes [82,83,84,85,86], which was delinquency in this case. This is in line with GST [29] and the life course perspective [87,88,89]. Very early childhood is not only a period of heightened vulnerability to adversity, but adversities that occur during this sensitive period of child development may be especially impactful on later delinquency [13]. The second aim of our study was to investigate whether multiple, criminologically relevant factors mediate the relationship between early ACEs and adolescent delinquency. More specifically, we explored whether child property delinquency, substance use, low self-control, unstructured socializing, and parent–child attachment at age 11 mediated the relationship between early ACEs and delinquency in adolescence. Overall, our findings indicate that child property delinquency and child low self-control were the most robust mediators, followed by child substance use. Taken together, these findings hold important implications for criminological theory and suggest that prior (i.e., early) delinquency (i.e., property delinquency or substance use) and self-control in middle childhood are likely central mechanisms in understanding involvement in delinquency for adolescents who were exposed to ACEs in early childhood.

Theorizing on development points to parenting early in life as a key mechanism of low self-control [40,90]. However, recent research suggests that exposure to multiple ACEs in early childhood may increase the risk of developmental challenges in children, including a reduction in self-control and elevated behavioral problems [38,39,40,45,46,47]. Relatedly, a distinct literature also details that self-control remains one of the most robust predictors of delinquency in childhood and beyond [43,44]. The findings from the current study unite these two streams of research and show that children with higher ACE exposure (i.e., higher exposure to stressors) tend to have lower levels of self-control, and in turn, self-control is a key intermediary mechanism that links ACE exposure and delinquency. 

Regarding the finding that prior delinquency and substance use in childhood also partially mediate the association between ACEs and adolescent delinquency, these findings provide support for theoretical frameworks that detail how early life engagement in delinquent behaviors can result in a continuity of behavior and escalation of delinquency into adolescence [37,91]. Accordingly, our findings expand upon previous research on the links between early exposure to adversity (e.g., abuse, neglect, household dysfunction and hazards, and food insecurity) and on early-onset delinquency and other behavioral problems [39,92,93,94] by demonstrating that the association between early ACEs and early-onset misconduct in turn can disrupt prosocial development and make it more likely that ACE-exposed individuals will engage in criminal activity in subsequent stages of the life course. 

Furthermore, unstructured socializing with peers and parental bonding measures in middle childhood had relatively weak, albeit mostly significant, mediating effects. Specifically, parent–child attachment had a weak effect on ACEs and adolescent delinquency, whereas child unstructured socializing weakly and inconsistently mediated the relationships between ACEs and delinquency, falling from significance in the case of three or more ACEs. These findings are somewhat surprising given the amount of literature pointing to low attachment to parents and unstructured socializing with friends as robust predictors of involvement in delinquency [48,49,50,62,95,96]. Still, our findings point to the need to simultaneously compare multiple relevant mediators. 

### 7.1. Limitations and Future Research 

There are several limitations to this study that warrant mention. First, the measures of ACEs were limited; there were three measures of ACEs included in the CDC-Kaiser ACE study that we were unable to capture in our study. Specifically, we were unable to measure childhood sexual abuse, physical neglect, and emotional neglect because there were no behavioral items on these forms of maltreatment in the UK MSC study. Moreover, there may be other ACEs not outlined in the original ACE study that may be important predictors of delinquency that we were unable to capture, such as the death of a loved one or parent, exposure to community violence, and exposure to natural disasters. Thus, future research should continue to explore the relationship between ACEs and adolescent delinquency by utilizing an even broader inventory of ACEs that goes beyond those examined here. Second, we were unable to continue examining these precise ACEs beyond age 7 due to data limitations. It is possible that by age 11 (S5) or 14 (S6), some children could have newly experienced ACEs that may shape delinquency at age 14. Subsequently, the current study may have underestimated the impact of ACEs on adolescent delinquency. Future prospective studies should seek to capture ACEs at various points of time during both early and middle childhood as well as early adolescence. Third, future research should explore how effects might vary by key demographics indicators, such as race/ethnicity, class, and sex. While post hoc analyses of MCS data do not yield evidence of moderation by these factors, some demographic categories are quite small (e.g., Black and mixed-race individuals made up about 3–5% of the sample). Future research should consider reexamining these relationships with more racially diverse samples. Finally, our study included a sample of children from the UK, which is not generalizable to other countries. Still, the UK sample is also a strength, since much of the work that exists on ACEs and adolescent delinquency has been conducted in the U.S.

Despite these limitations, our study offers two important strengths that make unique contributions to our understanding of adolescent delinquency. First, our study used longitudinal data from the UK to examine how ACEs in early childhood shape later involvement in delinquency. This approach allowed us to demonstrate the critical role ACEs play in later delinquency involvement, including how these processes are shaped by the accumulation of early ACE exposure. Second, our attention to the various ways the relationships between early ACEs and adolescent delinquency may be mediated by multiple theoretically relevant mechanisms during middle childhood is another strength, considering the lack of research in this area that investigates the middle childhood mechanisms that link ACEs to adolescent delinquency. 

### 7.2. Policy Implications 

Based on the current study’s findings, it is imperative that trauma prevention and intervention efforts should screen for ACEs, especially in early childhood. To reduce the detrimental effects of ACEs, prevention interventions should be applied directly after the adverse event. For example, psychological first aid (PFA) is often implemented in nonfamily settings, such as schools or health facilities, to address ACEs. PFA identities children and their caregivers immediately after a stressful life event and provides information, education, comfort, and support to hasten recovery and increase resiliency [97]. Research has shown that PFA has been successful in improving connectedness and stress among youth who have experienced traumatic life events, such as disaster, death of a family member, and serious injury [98], and some recent evidence suggests it may also be effective in the UK context [99], though more focus on children and adolescents is needed [100]. 

In recent years, screening for ACEs has been posited as a public health strategy by several health organizations not only in the US [101] but also in the UK [102,103,104] to improve the long-term health of children and their families. In the UK specifically, the government made a commitment in 2013 to explore the implementation of routine ACE screenings for adolescents and adults [105]. One of the key principles in public health screening approaches is that the screening practices have the most benefits when there are established interventions that may mitigate specific negative life outcomes—like delinquency [106]. Our study points to the importance of self-control, property delinquency, substance use, and to a weaker effect of unstructured socializing and parental bonding in middle childhood as central mechanisms in understanding the linkages between early ACEs and adolescent delinquency. Thus, intervention practices that seek to identify and address these important mechanisms may be best suited for alleviating the risk of adolescent delinquency. 

Considering the connection between ACEs, self-control, and adolescent delinquency, the findings also suggest that efforts to support the development of greater self-control during childhood, especially among individuals with higher ACE exposure, may be useful in reducing involvement in delinquent behavior. For example, a recent meta-analysis of nearly 50 randomized controlled trials demonstrated a range of applicable interventions including mindfulness training, family-based interventions, and social and personal skill development that can be used to improve self-regulation and in turn improve behavioral conduct [107].

## Figures and Tables

**Table 1:** Descriptive Statistics.

	*Mean/%*	*SD*	*Range*
* **Outcome Variable** *			
Adolescent Delinquency	0.75	1.20	0–11
* **Independent Variables** *			
Adverse Childhood Experiences: 0	38.09%	-	0–1
Adverse Childhood Experiences: 1	44.66%	-	0–1
Adverse Childhood Experiences: 2	14.34%	-	0–1
Adverse Childhood Experiences: 3+	2.91%	-	0–1
* **Covariates** *			
Age in Years	13.77	0.45	13–15
Male	49.25%	-	0–1
Race: White	79.90%	-	0–1
Race: Asian	10.91%	-	0–1
Race: Black	3.11%	-	0–1
Race: Mixed	4.71%	-	0–1
Race: Other	1.37%	-	0–1
Mother Age at Birth	29.10	5.76	13–51
Parent Education NVQ 1	7.31%	-	0–1
Parent Education: NVQ 2	27.05%	-	0–1
Parent Education: NVQ 3	14.58%	-	0–1
Parent Education: NVQ 4	31.60%	-	0–1
Parent Education: NVQ 5	4.66%	-	0–1
Parent Education: Other	3.35%	-	0–1
Parent Education: None	11.45%	-	0–1
Household Poverty	31.60%	-	0–1
Household Size	4.22	1.28	2–17
Low Neighborhood Safety	1.83	0.85	1–5
* **Mediators** *			
Child Property Delinquency	7.45%	-	0–1
Child Substance Use	12.54%	-	1–1
Child Low Self-Control	1.60	0.48	1–3
Child Unstructured Socializing	0.00	0.86	−2.45–1.17
Parent–Child Connectedness	0.49	0.62	0–3

**Table 2:** Unadjusted and Adjusted Negative Binomial Regression Models of the Association between Adverse Childhood Experiences and Adolescent Delinquency (*N* = 11,192).

	*Adolescent Delinquency*			
Variables	IRR	CI	IRR	CI
* **Adverse Childhood Experiences** *				
1	1.21 **	1.11–1.31	1.18 **	1.09–1.28
2	1.45 **	1.31–1.61	1.39 **	1.25–1.54
3+	1.66 **	1.39–2.00	1.47 **	1.24–1.76
* **Covariates** *				
Age in Years	-	-	1.22 **	1.12–1.32
Male	-	-	1.55 **	1.44–1.66
Race: Asian *(Ref: White)*	-	-	0.72 **	0.62–0.85
Race: Black *(Ref: White)*	-	-	1.11	0.92–1.33
Race: Mixed *(Ref: White)*	-	-	1.17 *	1.01–1.35
Race: Other *(Ref: White)*	-	-	1.18	0.86–1.61
Mother Age at Birth	-	-	0.98 **	0.97–0.99
Parent Education: NVQ 2 (S5) *(Ref: NVQ 1)*	-	-	1.09	0.95–1.25
Parent Education: NVQ 3 (S5) *(Ref: NVQ 1)*	-	-	0.91	0.78–1.07
Parent Education: NVQ 4 (S5) *(Ref: NVQ 1)*	-	-	1.04	0.90–1.19
Parent Education: NVQ 5 (S5) *(Ref: NVQ 1)*	-	-	1.19	0.97–1.46
Parent Education: Other (S5) *(Ref: NVQ 1)*	-	-	0.86	0.70–1.07
Parent Education: None (S5) *(Ref: NVQ 1)*	-	-	1.08	0.91–1.27
Household Poverty	-	-	1.14 **	1.05–1.25
Household Size	-	-	1.00	0.97–1.04
Low Neighborhood Safety	-	-	1.04	0.99–1.08

* *p* < 0.05; ** *p* < 0.01.

**Table 3:** KHB Test of Mediators between ACE Exposure and Adolescent Delinquency (*N* = 11,192).

	Adolescent Delinquency					
	*One ACE*		*Two ACEs*		*Three or More ACEs*	
*Mediators*	% Reduction	*z*-Score	% Reduction	*z*-Score	% Reduction	*z*-Score
Child Property Delinquency	13.46%	3.71 **	10.38%	4.48 **	20.39%	4.98 **
Child Substance Use	6.34%	2.01 *	10.53%	4.98 **	11.26%	3.76 **
Child Low Self-Control	16.49%	4.36 **	10.88%	4.39 **	11.46%	4.26 **
Child Unstructured Socializing	5.95%	2.05 *	4.78%	2.88 **	2.31%	1.11
Parent–Child Connectedness	8.25%	3.31 **	3.80%	3.19 **	3.04%	2.80 **
Total	**50.49%**	-	**40.37%**	-	**48.46%**	-

* *p* < 0.05; ** *p* < 0.01.

## Data Availability

Data used in the current study are available at: https://cls.ucl.ac.uk/cls-studies/millennium-cohort-study/.

## Appendix A

**Table A1:** Description of Adverse Childhood Experiences at Ages 5 and 7 ^a^.

- **Verbal Maltreatment (Ages 5 and 7)**—the main responder was questioned about ‘*How often shouts at child when naughty? Daily, often [about once a week or more], sometimes [once a month], rarely, or never*’. Dichotomized: daily/often *versus* sometimes/rarely/never [Reference Category]
- **Physical Maltreatment (Ages 5 and 7)**—the main responder was questioned about ‘*How often smacks the child when naughty? Daily, often [about once a week or more], sometimes [once a month], rarely or never*’. Dichotomized: daily/often *versus* sometimes/rarely/never [Reference Category]
- **Parental Divorce (Ages 5 and 7)**—the main responder was asked about marital status to identify occurrence of divorce or legal separation (‘*Divorced, legally separated, 1st marriage, remarried, 2nd or later married, single, never married, or widowed*’). Dichotomized: divorced/legally separated *versus* single/widowed/married [Reference Category]
- **Parental Mental Illness (Ages 5 and 7)**—Kessler 6 (K6) ^b^ scale was used to assess maternal mental health in the last month asking the responders how often they felt depressed, hopeless, restless or fidgety, worthless, or that everything was an effort. Respondents answered on a five-point scale from 1 (all the time) to 5 (none of the time). We reversed and rescaled all items from 0 to 4 for analysis purposes, so that high scores indicated high levels of psychological distress. We used validated cut offs for severe mental illness [‘*yes (scores >=13)/no*’]
- **High Frequency of Parental Alcohol Use (Ages 5 and 7)**—The main responder responded to a question about their usual frequency of alcohol consumption (‘*Every day, 5–6 times per week, 3–4 times per week, 1–2 times per week, 1–2 times per month, less than once a month, or never.*’). Dichotomized: every day/5–6 times per week *versus* 3–4 times per week/1–2 times per week/1–2 times per month/less than once a month/never [Reference Category]
- **Domestic Violence (Ages 5 and 7)**—The main responder was asked about the use of force by the partner in the relationship (‘*Yes, No*’) Dichotomized: Yes *versus* No.
- **Parental Drug Use (Age 5) ^c^**—The main responder was asked about the use of recreational drugs in the past 12 months (‘*regularly, occasionally, cannot define, or never*’). Dichotomized: regularly *versus* occasionally/cannot define/never [Reference Category]

^a^ Adapted from [70]. ~98% of main respondents were mothers; ^b^ [108]; ^c^ This item was not assessed at age 7 (S4) as it was not available.
